# Killifish switch towards mammalian-like regeneration upon aging

**DOI:** 10.18632/aging.203995

**Published:** 2022-03-31

**Authors:** Sophie Vanhunsel, Steven Bergmans, Lieve Moons

**Affiliations:** 1 Neural Circuit Development and Regeneration Research Group, Animal Physiology and Neurobiology Division, Department of Biology, KU Leuven, Leuven 3000, Belgium; 2 Leuven Brain Institute, Leuven 3000 , Belgium

**Keywords:** central nervous system, aging hallmarks, axonal regeneration, African turquoise killifish, short lifespan

In our aging society, the prevalence of brain trauma and age-related neurodisorders, in which neurons are damaged and/or lost, has been increasing. Unfortunately, spontaneous recovery from these conditions is not occurring in humans, as both neuron-intrinsic and -extrinsic factors hinder regeneration in the adult mammalian central nervous system (CNS). One way to discover novel targets to promote CNS repair, is to use model organisms that are capable of restoring damage in their adult CNS. Unlike mammals, several teleost species retain their neuroregenerative ability after birth, and can successfully replace lost neurons and regrow severed axons throughout adulthood. This has resulted in fish models becoming increasingly popular to study injury-induced CNS recovery, and to help deciphering the cellular and molecular mechanisms that are required for functional circuit restoration in the adult mammalian brain. Indeed, despite the difference in repair capacity between fish and mammals, many molecules and processes that regulate neuroregeneration are conserved within vertebrate species, and findings obtained in fish are considered transferable to humans [1]. How aging impacts the fish’s ability to functionally recover from CNS damage, however, has only been limitedly studied up till now.

**Figure 1:**
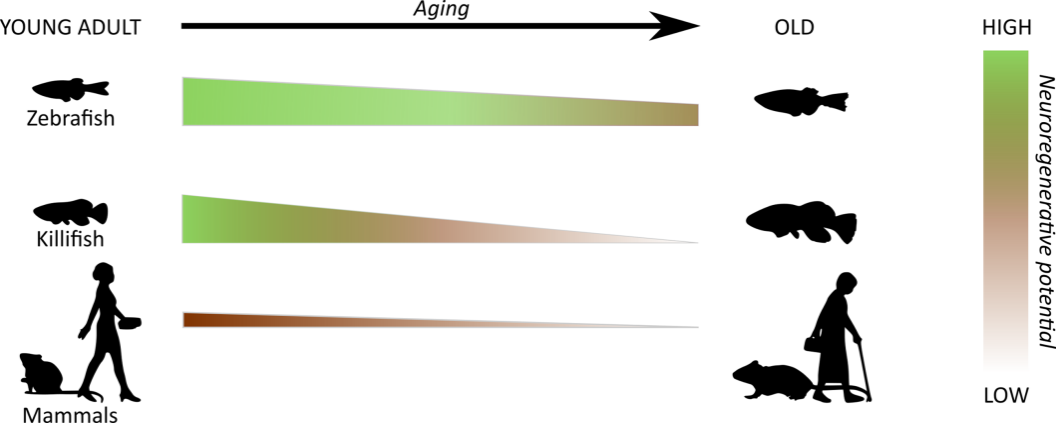
**Differences in regenerative ability of the central nervous system through species and aging.** In contrast to zebrafish that maintain their neuroregenerative capacity throughout adulthood, killifish completely lose this ability upon aging and do not functionally recover from central nervous system damage. As such, with age, the regenerative response of the killifish central nervous system switches to one that resembles (young) adult mammals.

Over the recent years, the fast-aging African turquoise killifish (*Nothobranchius furzeri*) has emerged as an excellent biogerontology model, as it uniquely combines (i) the advantages of classic fish species with (ii) a lifecycle comparable to that of invertebrate models. Its explosive growth *post* hatching, early sexual maturation and short lifespan are the result of the habitat in which this fish resides, namely temporary freshwater pools in Africa. Importantly, despite having a lifecycle of only a few months, killifish do age. They even age in a similar way as humans, presenting many of the well-described aging hallmarks, yet often magnified and occurring within a much shorter time frame. Interestingly, killifish appear to pay a price for their fast growth and aging. In contrast to zebrafish –that maintain their neuroreparative ability *albeit* regenerate less efficiently at old age [2–4] –, killifish completely lose their regeneration capacity at old age and are unable to fully recover from CNS injury [5,6]. Using an optic nerve crush injury model in killifish of different ages, we indeed revealed that, in contrast to young fish, aged animals do not regain vision following damage. An inadequate intrinsic capacity of aged retinal ganglion cells (RGCs) to revert to a “regenerative state” as well as a growth-inhibiting neuron-extrinsic environment seem to contribute to this impairment [6], similar to what has been described for (young) adult mammals. We postulate that age-associated changes within neurons and their glial environment –already manifesting before damage occurs– negatively affect the regeneration potential of the killifish CNS, which then leads to a mammalian-like regenerative response upon injury. Indeed, recent findings from our team revealed the presence of several aging hallmarks particularly in the old killifish visual system [7], which could underlie the flawed optic nerve regeneration that is observed in aged killifish. With increasing age, we revealed reduced expression levels of growth-associated genes in retinal neurons, thereby affecting the intrinsic ability of RGCs to regrow their axons. Additionally, oxidative stress was shown to pile up in the aged killifish retina and tectum, which is known to lead to mitochondrial dysfunction and therefore very likely contributes to failure of the energy-demanding regenerative process. Next to neuron-intrinsic changes, we observed signs of astrogliosis, inflammaging and a senescence-associated secretory phenotype upon aging, which might sensitize the old killifish CNS and result in growth-unfavorable glial reactivity upon injury. An increased glial reactivity and inflammation is known to occur upon CNS injury in young fish, yet these processes are transient and therefore assumed to not persist long enough to create a disadvantageous environment for repair. In fact, these acute glial responses are even believed to be beneficial for CNS recovery. The onset of astrogliosis and a chronic inflammatory status in the killifish CNS during physiological aging seems to result in a more extensive and extended glial reactivity upon nerve injury, which is known to be detrimental for regeneration in mammals. Strikingly, the exaggerated neuroinflammatory events then result in the formation of a long-term glial scar, which has never been shown in any other regeneration-competent model before. Our killiteam at KU Leuven is the first to reveal evidence for such glial scarring within the CNS of aged killifish when subjected to either optic nerve crush or stab injury in the telencephalon [5,6]. This suggests that, in addition to intrinsic factors and altered glial responses, neurorepair in the old killifish CNS is hindered chemically as well as mechanically by glial scar tissue. While resembling zebrafish at young age, aged killifish thus seem to mimic (young) adult mammals, and position themselves in-between zebrafish and mammals regarding their regenerative potential throughout adulthood (Figure 1).

In summary, it seems that explosive growth and/or fast aging eventually turns the killifish CNS into a regeneration-incompetent organ, which has not been reported before in any teleost species. By shifting its regenerative potential from high to low with increasing age and forming a glial scar following CNS injury, the killifish puts itself in the exceptional position of resembling (young) adult mammals when at old age. Having these mammalian-like characteristics, together with sharing many of their protein coding genes with humans [5,8], the killifish offers unique opportunities to identify regulators of neuroregeneration, with high translational value to humans. Using the killifish might thus open a new door towards the development of efficient regenerative therapies for the aged, injured or diseased mammalian CNS.
